# Study on the process of cardiomyocyte apoptosis after pulsed field ablation

**DOI:** 10.3389/fcvm.2023.1112131

**Published:** 2023-04-05

**Authors:** Shaobo Fan, Fenglin Jia, Yingjian Cui, Dongyan Wu, Le He, Fan Zhang, Zhixiao Xue, Xinyu Xu, Fengmin Lu, Wei Ma, Siying Su, Yanpeng Chen, Zhenxu Liu

**Affiliations:** ^1^ Department of Cardiology, Chest Hospital, Tianjin University, Tianjin, China; ^2^ School of Biomedical Engineering and Technology, Tianjin Medical University, Tianjin, China; ^3^ Department of Research and Development, Tianjin Intelligent Health Medical Co., Ltd, Tianjin, China

**Keywords:** atrial fibrillation, pulse field ablation, pulmonary vein isolation, cardiac ablation, myocardiocyte

## Abstract

**Background:**

The development of pulsed field ablation (PFA) as a new technique for pulmonary vein isolation (PVI) has been advancing rapidly in recent years. My team's previous work has shown the safety and long-term efficacy of bipolar asymmetric pulses in animal experiments. However, in ongoing clinical trials, we have observed that atrial fibrillation (AF) recurs in some patients after surgery, but the rhythm returns to normal without surgical intervention after seven days, and there is no recurrence in the follow-up.Based on this observation, we have proposed the hypothesis that myocardial cell apoptosis may play a role in AF recurrence after PFA. Our team has designed animal experiments to verify this hypothesis and further investigate the process of PFA-induced cardiomyocyte apoptosis.

**Methods:**

Pulse field ablation was performed on 15 dogs and the animals were dissected at various time points after the operation (immediately, 3 days, 7 days, 30 days, and 150 days). To obtain ablation voltage maps, electroanatomic mapping was performed before and after ablation and before dissection. The ablation area was also subjected to HE and TUNEL staining to analyze apoptosis and pathological results.

**Results:**

The edge area of the ablation in the pulmonary vein (PV) demonstrated continuous dynamic changes from 0 to 2 h after the operation and a slight expansion of the ablation range was observed in the long-term follow-up. Myocardial intima hyperplasia was observed from 0 to 7 days. Local apoptosis was detected from 0 to 2 h and massive, concentrated apoptosis was observed at 3 days. No recurrence of apoptosis was seen at 7 days, 30 days, and 150 days.

**Conclusions:**

The results of this study showed that after pulse field ablation (PFA), the central ablation area of the canine heart experienced immediate cardiomyocyte death. Meanwhile, cardiomyocytes in the edge ablation area underwent apoptosis, which began from 0 to 2 h post-operation and ended between 3 and 7 days. This process occurred simultaneously with intimal thickening.In the long-term follow-up group, there was no recovery of isolation and no recurrence of cardiomyocyte apoptosis, and no change was observed in the endomyocardial intima.

## Introduction

Our previous research has verified the safety and effectiveness of two-phase asymmetric pulse field ablation (PFA) through cell and animal experiments. The results showed that PFA can produce long-term, effective isolation of pulmonary veins (PVs) using our specified ablation parameters (1, 2). However, during an ongoing clinical trial, it was observed that some patients experienced acute atrial fibrillation (AF) recurrence within 1 to 3 days after PFA. Despite this, AF healed spontaneously after a week and there was no recurrence within three months of follow-up.To explain this phenomenon, we reviewed relevant literature and hypothesized that it may be due to differences in cell death time caused by different types of electroporation(EP) resulting from PFA (3–11). In the central ablation area close to the electrode, high field strength and current density result in immediate cardiomyocyte death. However, in the edge of ablation area with weaker field strength and current density, EP is insufficient to cause immediate cell death and cardiomyocytes die gradually through apoptosis.Immediately after PFA, all ablation sites experience EP and cell membrane damage, leading to pulmonary vein isolation(PVI) observed through mapping. The recovery of the transmembrane potential in non-dead myocardial cells leads to AF recurrence one day after surgery. However, as the myocardial cells that did not die immediately undergo gradual apoptosis, AF gradually heals and does not recur. To further study the development of cardiomyocyte death after PFA and construct a theoretical model of the process of myocardial cell death., we designed an animal experiment.

## Materials and methods

### Materials

#### Experimental animals

Fifteen healthy Labrador retrievers (weight 25 ± 3 kg; of either sex) were provided by Beijing Tonghe Litai Biotechnology Co., Ltd. [SCXK (Jing) 2019-0005]. All our experiments were approved by the Ethics Committee.

#### Reagents and instruments

The medicine used in the present study were Sumianxin II injection [compound preparation of Jingsongling, edetic acid, Dihydroetorphine hydrochloride (DHE), and haloperidol] (Jilin Dunhua Shengda Animal Pharmaceutical Co., Ltd.), 1% propofol injection (AstraZeneca, Italy), and enoxaparin sodium injection (Sanofi, France).

**Figure 1:**
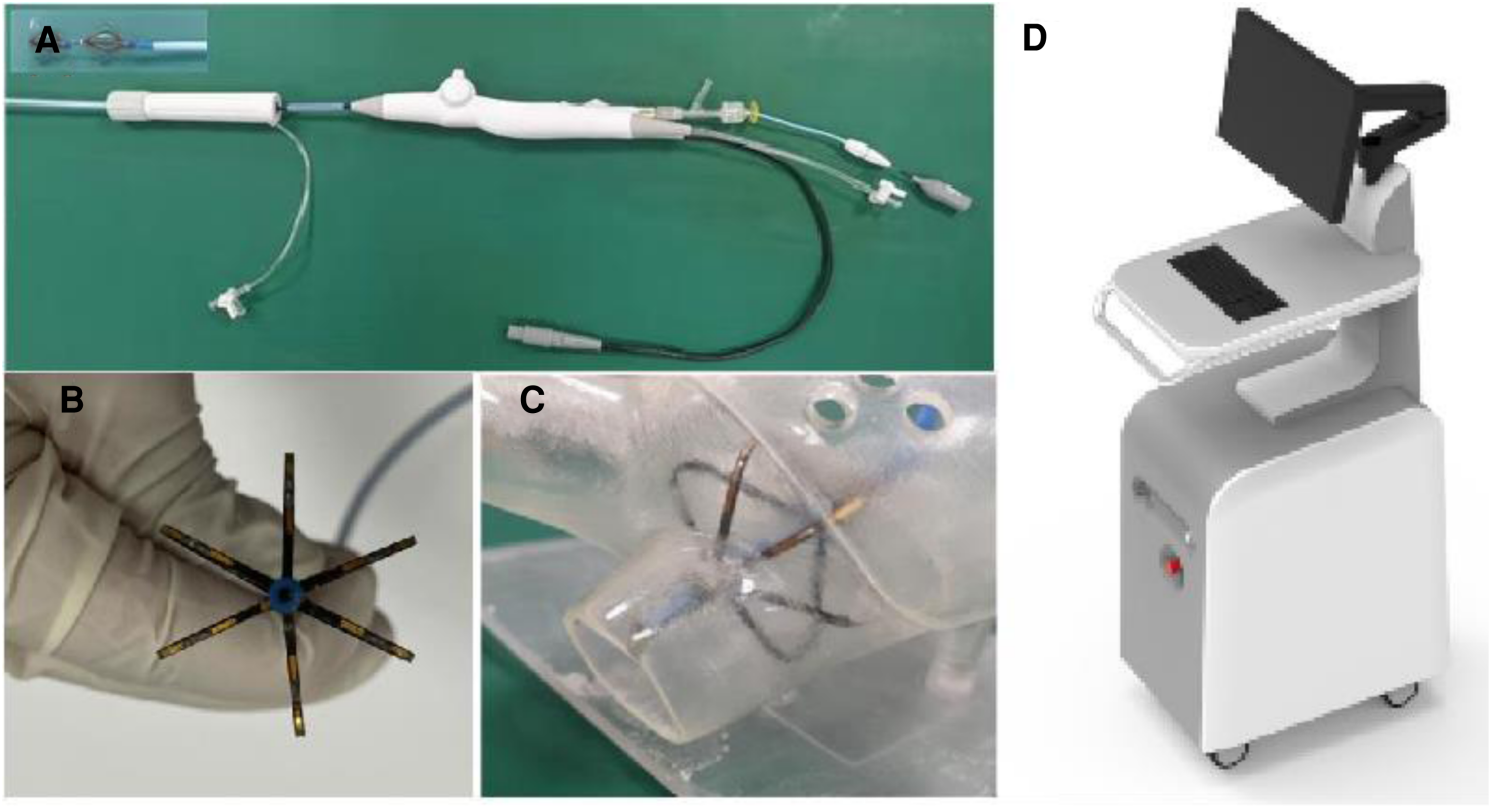
PFA system, (**A**) the ablation catheter, (**B**) the electrode part at the end of the ablation catheter, (**C**) the schematic diagram of the catheter placed in the left atrium model, and (**D**) the PFA pulse generator.

PFA system used in the experiment is provided by Tianjin Intelligent Health Technology Co., Ltd. The system includes a pulse field voltage generator and a 10.5F pulse ablation catheter (Figure 1A). The catheter consists of a handle, a tail end, and electrodes. The handle end of the catheter is equipped with a knob to control the shape, forward extension, or backward contraction of the skeleton for placement and electrode attachment. The catheter also features a hollow interior, allowing for drug injection or wire delivery. The head end of the catheter is equipped with six ablation electrodes, with a maximum diameter of 28 mm when fully opened. The PFA generator (Figure 1B) has a built-in power supply, charging, discharge, protection, emergency stop, and acquisition modules and can release a 500–1500 V microsecond pulse voltage. The animal experiment has confirmed the safety and effectiveness of the PFA system and it has passed various safety regulations. So far, numerous clinical trials have been conducted with the PFA system.

### Experimental method

#### Experimental design

In this study, we aimed to investigate the development of cardiomyocyte apoptosis after PFA. The experimental group was divided into two groups: short-term group A (6 dogs labeled A1 to A6) and long-term group B (9 dogs labeled B1 to B9). The same PFA catheter and effective ablation parameters used in clinical trials were applied to both groups. Group A underwent electroanatomic mapping before the operation and at 0.5, 1, 2 h post-operation and before dissection. Group B underwent mapping before the operation and immediately after the operation and before dissection. The animals were euthanized and dissected at day 0 (A1, A2), day 3 (A3, A4), day 7 (A5, A6), day 30 (B1, B2), and day 150 (B3 to B9) after the ablation procedure, and the results were analyzed through histological investigation (Table 1).

**Table 1:** Experimental grouping.

	Group A (short-term group, 6 dogs)			Group B (long-term group, 9 dogs)	
Euthanasia time(After the operation)	Day 0	Day 3	Day 7	Day 30	Day 150
Animal number	A1, A2	A3, A4	A5, A6	B1, B2	B3–B9
Electroanatomic mapping performed	before operation, immediately \ half an hour \ one hour \ two hours after operation and before dissection			before operation, immediately after operation and before dissection	

### Procedural details

#### Before operation

**Figure 2:**
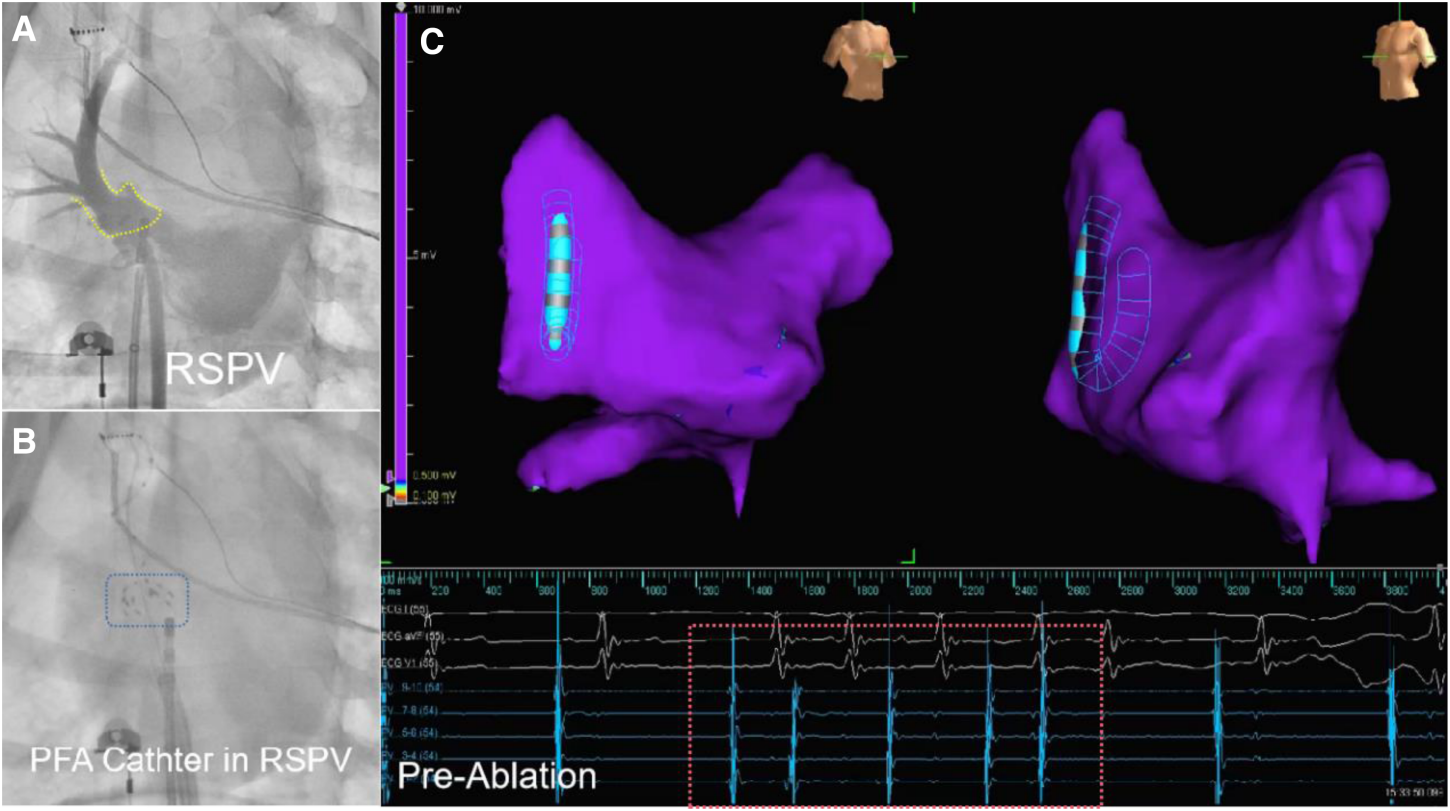
The DSA image and electroanatomical mapping (image from dog B2) is presented in three parts. Part (**A**) shows a DSA of the PV with the yellow dashed frame marking the PV ostium. Part (**B**) shows the PFA ablation catheter in preparation for discharge at the right superior PV antrum, with the blue dashed box showing the developing image of PFA ablation catheter under DSA. Part (**C**) shows that the left atrial electroanatomical modeling before ablation reveals an unisolated state of the PV. The red dashed box portion represents the pre-ablation PV electrogram, indicating that PV signals can be transmitted.

Before the operation, aspirin (5 mg/kg) was administered to the animals once daily for three days (Figure 2). The animals were fasted for 12 h and water was withheld for 6 h. Blood was collected from the precaval vein for preoperative routine blood testing. To induce anesthesia, the animals were given conventional doses of xylazine and midazolam. After establishing peripheral venous access, propofol was intravenously infused in a dose of 30–50 mg as needed to achieve stable anesthesia. The animals were placed in a supine position in custom U-shaped troughs on a surgical bed with digital subtraction angiography (DSA) support. Endotracheal intubation was performed and mechanical ventilation was provided with a ventilator. The skin on the anterior chest and bilateral inguinal areas was prepared for surgery by cleaning.

#### During operation

Throughout the procedure, the vital signs of the animals were monitored regularly. The surgical process was similar to the standard clinical practice. The Seldinger method was used to access the bilateral femoral veins and insert a 6F vascular sheath. The atrial septum sheath was then placed into the left atrium using intracavitary ultrasound and x-ray fluoroscopy. Heparin (6,000 units) was injected, with additional doses of 1,000 units every hour during the operation. After adjusting the position of the sheath, pulmonary venography was conducted to visualize the shape and branches of the PVs. An electroanatomic map of the left atrium was created using a circular mapping catheter. The semi-opened PFA ablation catheter was guided into the left atrium and positioned close to the target PV ostia. The ablation electrode was placed near the antrum of the PV to deliver the ablation pulses. The ablation site was then mapped to observe and evaluate the changes in the ablation range over time through voltage mapping comparisons (Figure 2).

#### After operation

All animals underwent a contrast-enhanced CT angiography to assess for any adverse conditions such as air emboli, thrombi, tears in the vascular access, and cardiac tamponade. The puncture sites were monitored after the catheter was removed. The animals' vital signs, behavior, and activity were observed after they awoke from anesthesia. Animals were injected with 20 IU/kg of intramuscular penicillin sodium + 0.9% normal saline twice daily for 3 days to prevent infection. Intensive care was provided after the operation, and any clinical changes were recorded.

**Figure 3:**
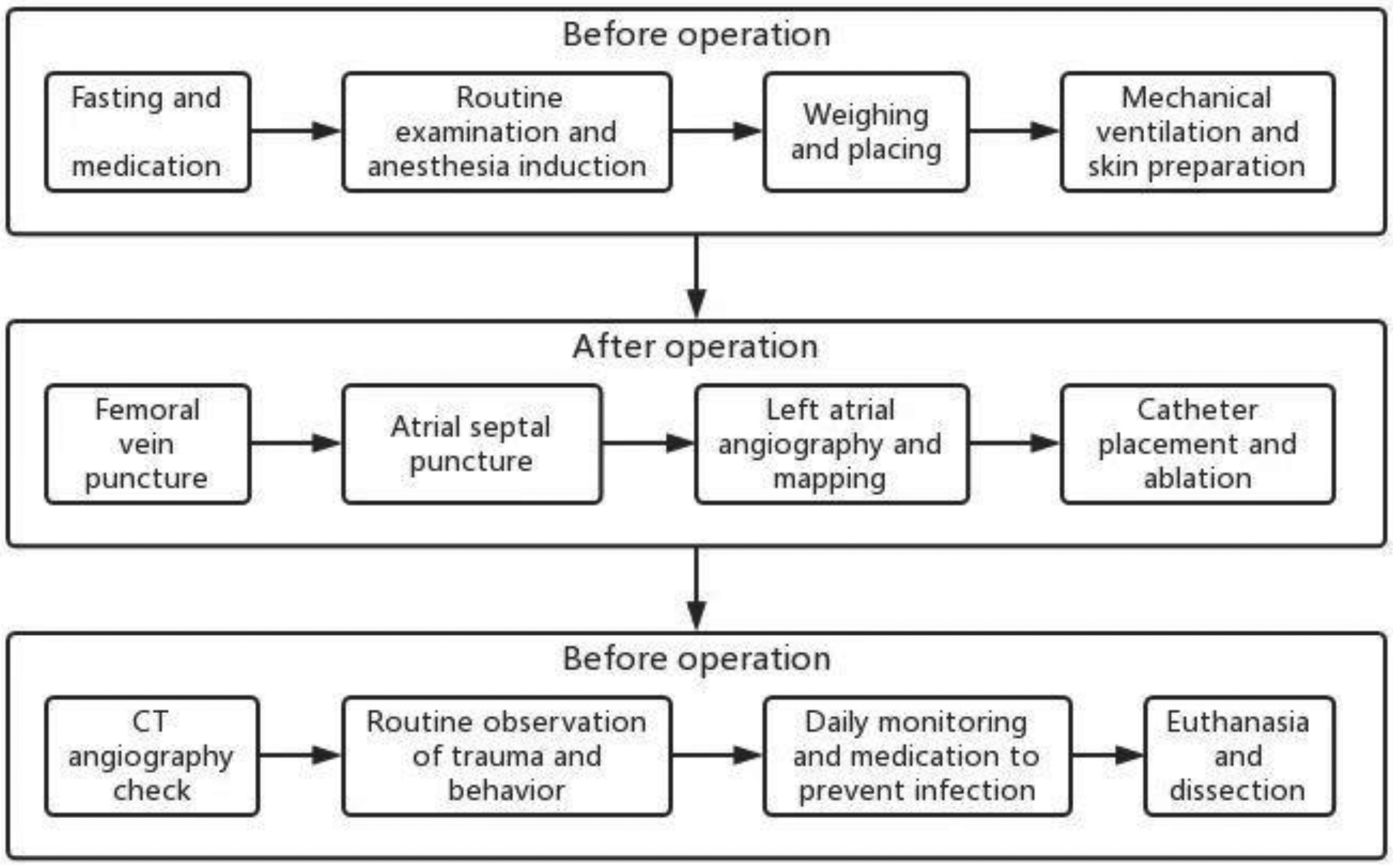
Procedure process.

The animals were euthanized by bloodletting on the specified days for general observation and pathological analysis. This process involved transecting the femoral triangle, cutting the femoral artery and vein to allow for blood to flow out, and flushing the bleeding parts with water to prevent coagulation. The animal died a few minutes later (Figure 3).

### Histological investigation

After the animals were euthanized, the hearts were dissected to examine the ablation site, and tissue samples were taken from the site and surrounding areas. The samples were then fixed in formalin and stained with HE (Hematoxylin and Eosin) and TUNEL(Terminal deoxynucleotidyl transferase dUTP Nick End Labeling). The results of the staining were examined under a microscope to assess changes in cardiomyocyte thickness, fibrosis, and apoptosis. The correlation of cell morphology, lesion depth, and apoptosis were analyzed by observing the HE and TUNEL staining. Quantitative data analysis of the TUNEL staining results from the short-term group was performed using ImageJ software to better understand changes in apoptosis. Two sections of tissue were selected from each animal for analysis. The apoptosis rate was calculated as the ratio of the positive area to the total area.

### Statistical analysis

The SPSS version 20.0 software was used for data analysis. A t-test was used to compare the measurement data between groups. *P *< 0.05 was defined as statistically significant.

## Results

### Clinical observation and survival rate

During the procedure, none of the 15 animals that received PFA displayed any noticeable arrhythmias or discomfort, with only minor muscle contractions observed. The animals quickly regained consciousness within 30 min post-operation and exhibited normal behavior, mental state, and appetite in the first day following the procedure. Throughout the experiment, there were no signs of depression, hair loss, diarrhea, or nasal bleeding in any of the animals. All animals successfully made it to euthanasia without any issues.

### Acute experiments

**Figure 4:**
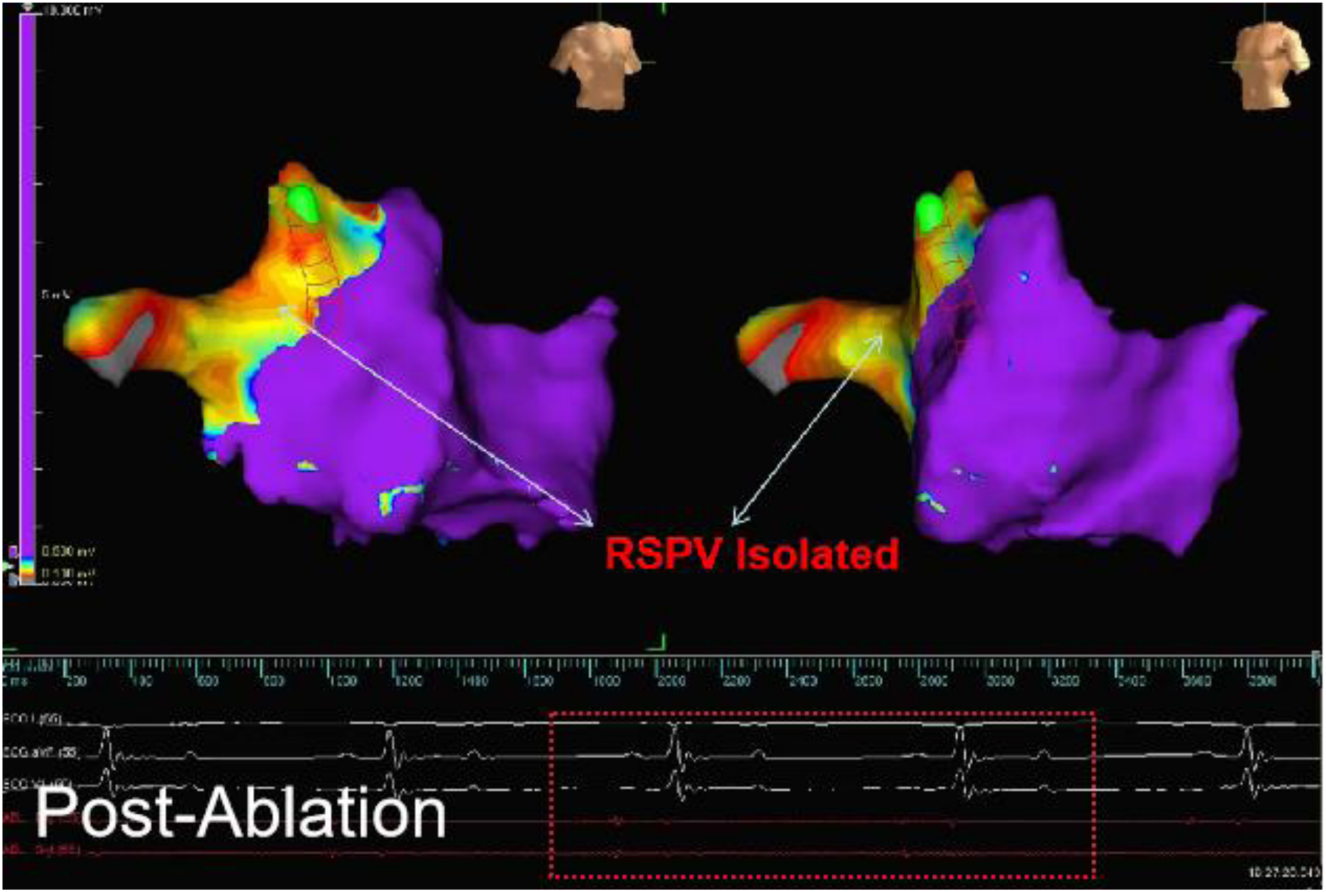
The electroanatomical mapping results immediately after surgery (image from dog B2) showed that the left atrial voltage mapping revealed low voltage in the right superior PV (RSPV). The red line frame represents the PV electrical signal after ablation, and the absence of far-field potential from the left atrium in PV indicates successful afferent block.

The ablation site was the superior PV, the acute success rate of ablation was 100% (15/15), and the mean energy delivery time was 21.6 s (mean time in group A: 19.833 s, mean time in group B: 22.777 s). All ablated PVs presented low voltage, indicating exit block, as assessed immediately after electroanatomic mapping (Figure 4).

### Remap results

**Figure 5:**
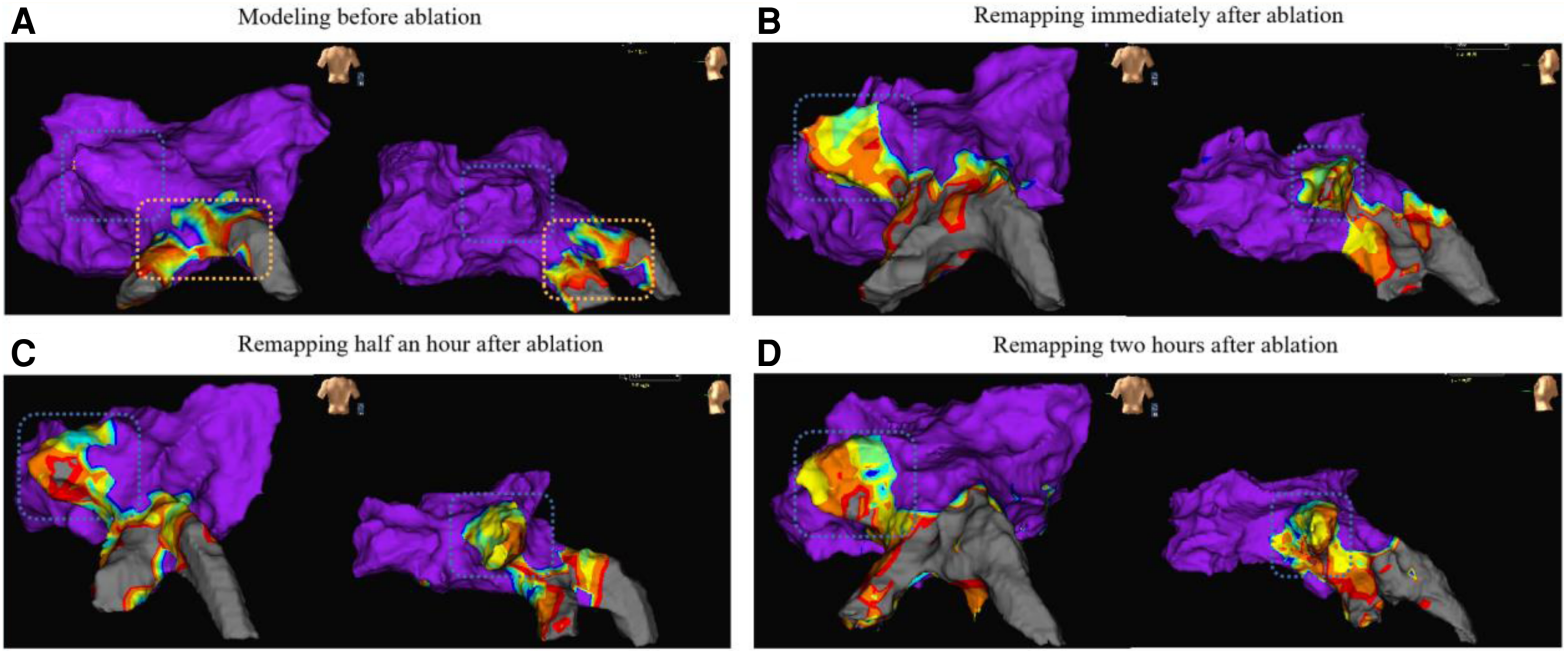
The remapping results (from dog A5) of the voltage maps are presented in anterior-posterior view (left side of each map) and left-anterior oblique view (right side of each map). Map A shows the preoperative left atrial modeling, Map B shows the voltage map immediately after ablation, Map C shows the voltage map 30 min after ablation, and Map D shows the voltage map 2 h after ablation. The blue wireframe in the figure represents the ablation site (left superior PV). The yellow frame in Map A partially represents the left inferior PV and its branches, and preoperative mapping revealed low voltage due to the animal itself.

The results of the electroanatomic mapping potential maps showed that PFA created a continuous and complete isolation zone (Figures 5A,B). The edges of the ablation area showed a tendency to shrink 30 min after surgery, while the ablation area was significantly larger two hours after surgery compared to its size immediately after surgery and 30 min after surgery. (Figures 5B–D).

**Figure 6:**
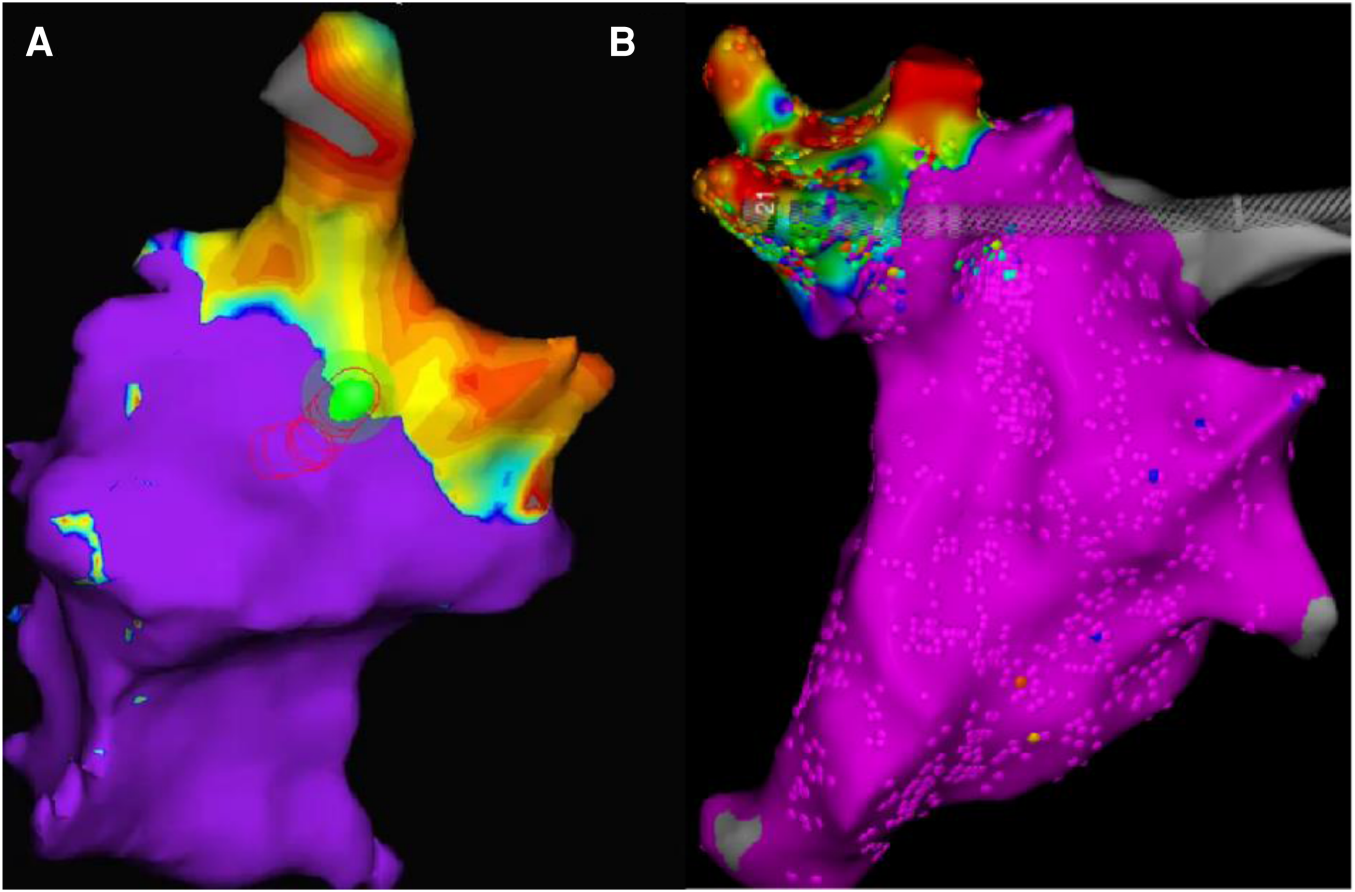
(results from dogB2, ablation site: right superior PV). The two maps have different color schemes due to the different systems used. The orientation and size of the pictures have been slightly adjusted for improved viewing, but the relative position and size remain unchanged. Map A displays the mapping results immediately after surgery, with the non-blue parts representing low voltage regions. Map B displays the mapping results 30 days after surgery, with the non-purple parts indicating low voltage regions.

In the long-term group(group B) of animals, the electroanatomic mapping results immediately after surgery and before dissection showed either a slight expansion or unchanged conditions. (Figure 6).

The results of voltage mapping showed that the postoperative potential changed over time. In the short-term (within a few hours), the potential at the edge sites of the ablation area showed a trend of reduction and expansion. However, the central area of the ablation range showed no change. In the long-term (more than 7 days), the ablation range tended to be stable, showing no change or further expansion. The changes observed in the voltage mapping data were further analyzed through histological staining analysis to understand the underlying reasons.

### Histological investigation

At necropsy after euthanasia, no injuries were observed in the trachea, esophagus, nerves, or pulmonary veins of any of the animals. Only a small amount of thrombus was observed in group A3, which was considered to be an organic injury. No thrombus was observed in the other groups. After ablation, the ablation site in the short-term group (A) appeared pale in color compared to the surrounding tissue, with a clear boundary. There was no significant difference in color between the ablation site and surrounding tissue in the long-term group (B).

**Figure 7:**
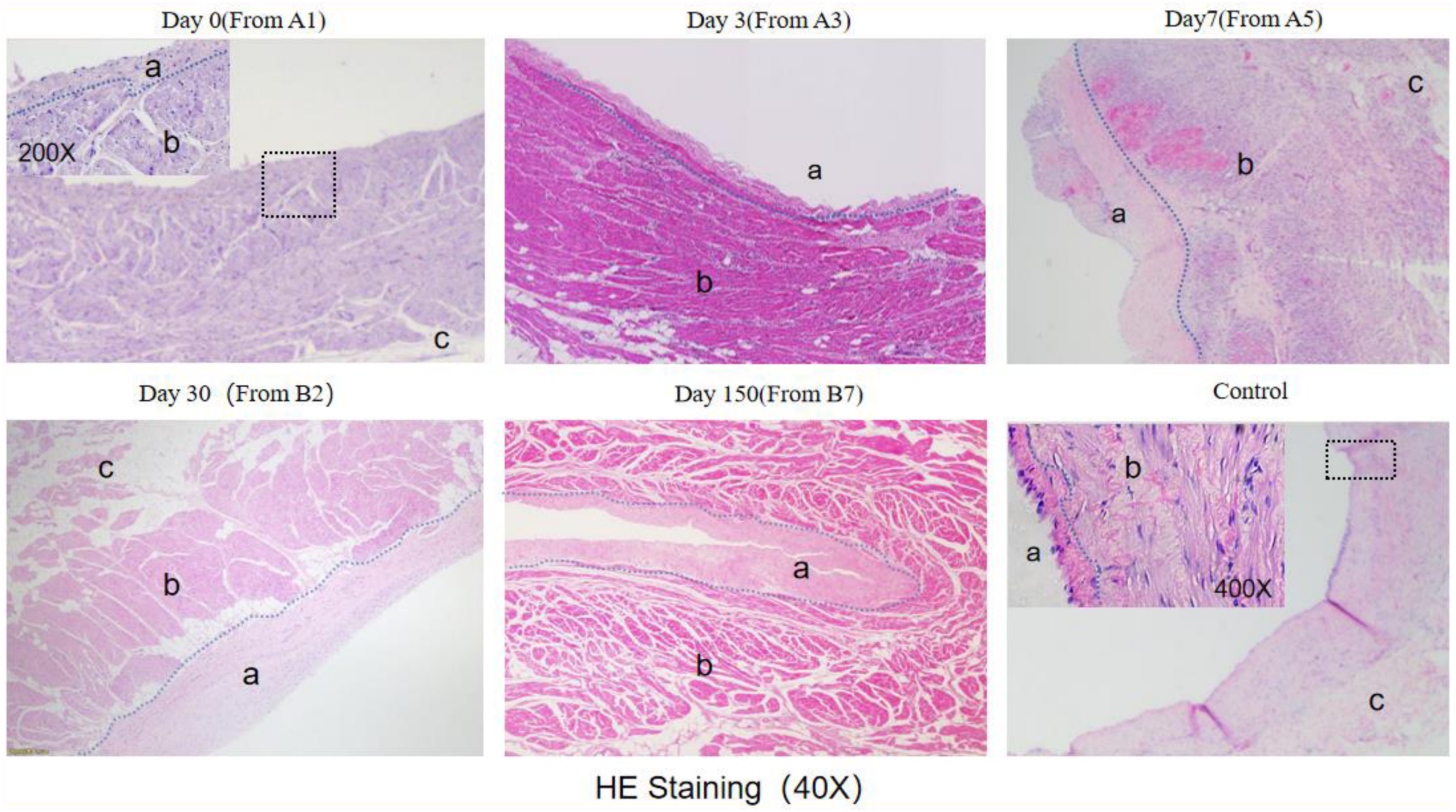
He staining results (observed under 40X microscope).The blue dashed line indicates the boundary between the endomyocardial intima and myocardial cells, with "a" marking the endomyocardial region, "b" marking the myocardial cell region, and "c" marking the epicardial region. The endomyocardial region in both the Day0 and control groups is small and difficult to distinguish under 40X magnification. The black wireframe region in the Day0 figure was observed under 200X magnification, while the black wireframe region in the control group was viewed under 400X magnification. The junction between the endomyocardial intima and myocardial cells is highlighted with a blue dashed line.

Histological analysis with HE staining revealed that the intima was thickened after ablation. Compared to the control group, the intima layer in the Day0 group was thicker. The thickening of the intima layer was more pronounced in the Day3 group and reached its maximum in the Day7 group. There was no significant change in the intima layer in the Day30 and Day150 groups compared to Day7 (Figure 7).

**Figure 8:**
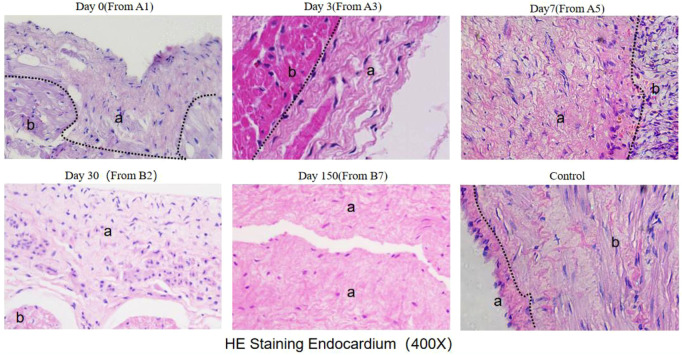
He staining results (observed under 400X microscope) The black dotted line marks the boundary between myocardial intima and myocardial cells. a indicates the intima layer, and b indicates the myocardial layer.

HE staining showed that the number of nucleated cells around cardiac myocytes increased at 3 days, reached the peak at 7 days, and disappeared at 30 days (Figure 8).

**Figure 9:**
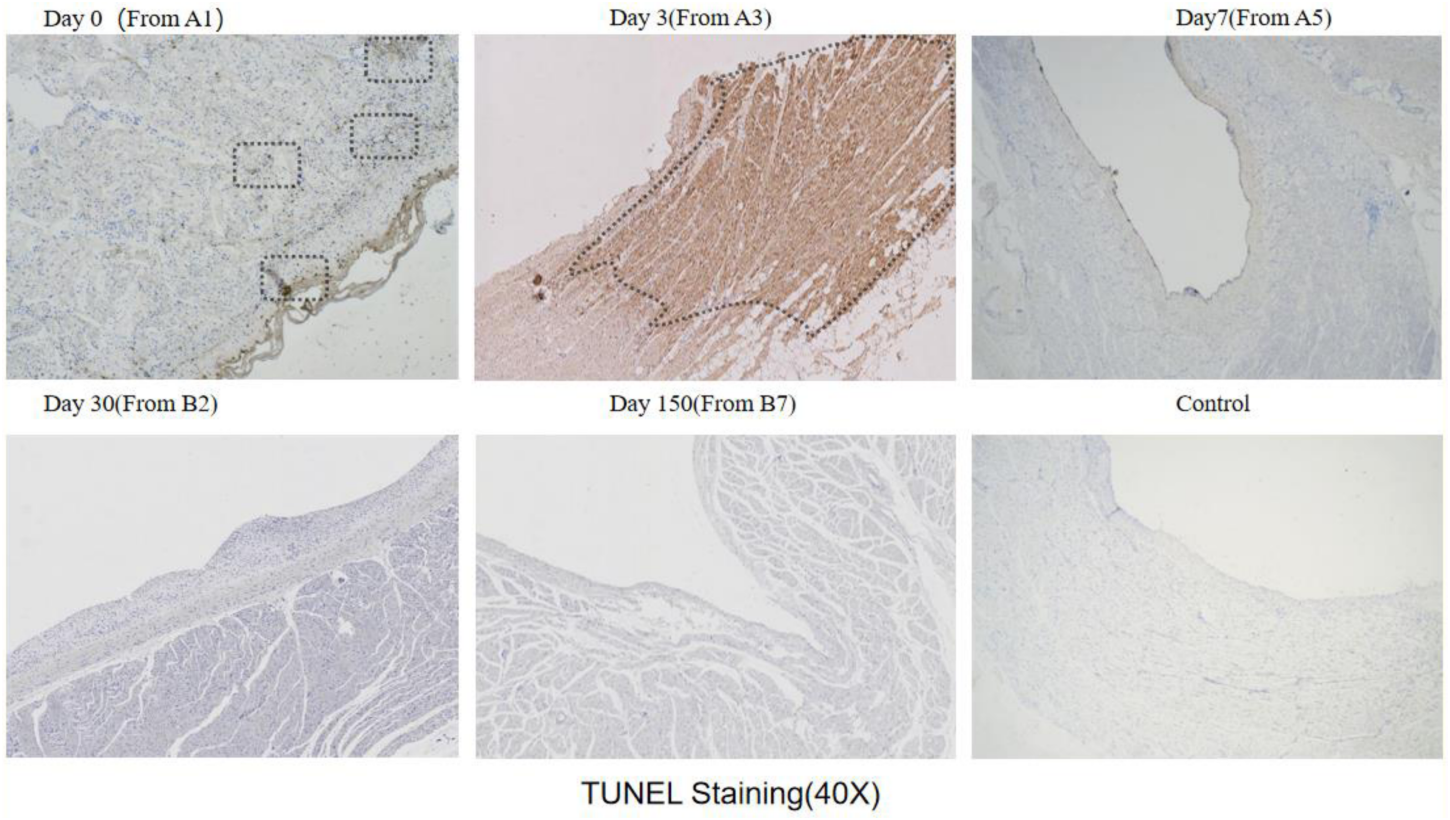
Tunel staining results (observed under 40x microscope): Tunel staining will darken apoptotic cells to brown or reddish brown, while ordinary cells will be blue or light; The black wireframe is dark apoptotic area, and Day3 and Day0 (a small amount) and Day3 have dark areas.

The results of TUNEL staining showed that after ablation, a minimal amount of localized apoptosis appeared in the Day0 group within the first two hours. In contrast, a significant concentration of apoptosis was observed in the Day3 group. However, no apoptotic cells were detected in the Day7, Day30, and Day150 groups (Figure 9).

The quantitative calculation results of Tunel staining showed that the average apoptotic ratio of Day0 group was 1.5864 ± 0.3290%, Day3 group was 36.5219 ± 0.8615% (*P* < 0.01), and Day7 group was 0.8615 ± 0.1694% (*P* < 0.01) (Table 2).

**Table 2:** Apoptosis rate.

Euthanasia time/d	Animal number	Apoptosis ratio	Average value	SD	*P*-0
Day 0	A1	1.8243%	1.5864%	0.3290%	
	A1	1.9088%			
	A2	1.3657%			
	A2	1.2467%			
Day 3	A3	36.6592%	36.5219%	0.8615%	2.39167 × 10^−6^
	A3	36.2792%			
	A4	37.6097%			
	A4	35.5394%			
Day 7	A5	0.2155%	0.3899%	0.1694%	0.004626517
	A5	0.4557%			
	A6	0.5951%			
	A6	0.2935%			

### Statistical summary and result analysis

The results of electroanatomic mapping indicated that the ablation area underwent continuous changes in the first two hours after the procedure. In the long term, it was found that the isolation remained effective, with no reduction in the ablation range.

The thickening of the endomyocardium started in the first two hours after ablation, reaching a gradual stabilization from three to seven days, with no significant changes observed at 30 and 150 days.

Similarly, apoptosis initiated in the first two hours after the procedure and came to an end from three to seven days. There was no reoccurrence of apoptosis at 30 and 150 days after ablation.

## Conclusions

Following PFA in canines, immediate death of cardiomyocytes occurred in the central ablation zone and apoptotic death gradually took place in the peripheral ablation zone. The process of massive cardiomyocyte apoptosis began in the first 2 h and was completed by 3 to 7 days, while thickening of the myocardial intima and apoptosis of myocardial cells progressed in parallel. The long-term outcomes indicated that the isolation remained effective, the ablation area did not shrink, and no reoccurrence of myocardial cell apoptosis was observed. Additionally, the myocardial intima remained unchanged.

## Discussion

**Figure 10:**
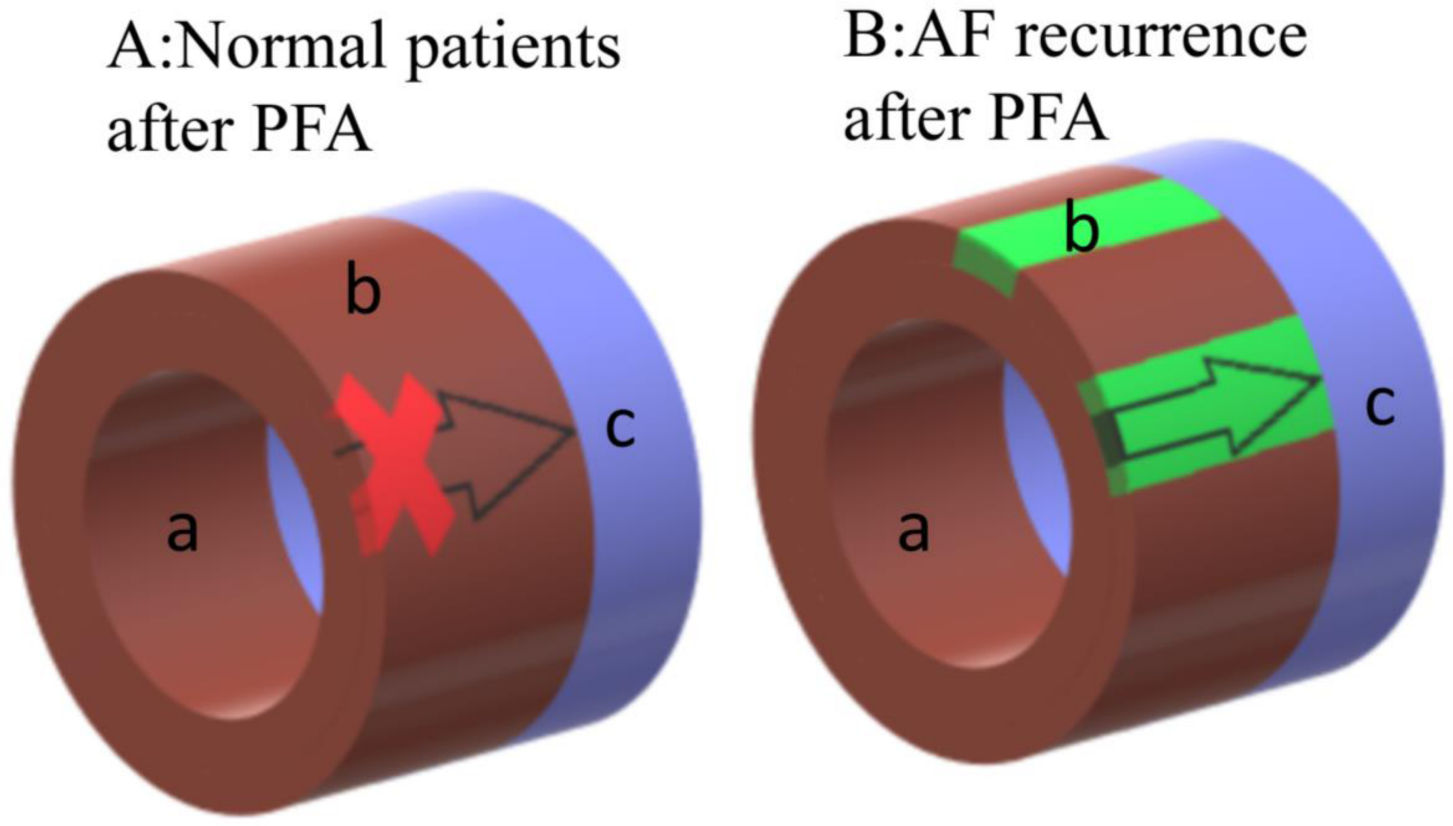
Schematic diagram of the ablation area after ablation.a indicates the endocardial membrane closing to the ablation electrode, which is the ablation central ablation area, and the myocardial cells in this area die instantly. b indicates the cardiomyocytes closing to the epicardium, which is the edge of ablation area, normal ablation also causes immediate death. However in some patients, this area that doesn't die immediately undergoes gradual apoptosis. c indicates the pulmonary vein. Figure A represents the normal schematic diagram after PFA, the PV electrical signal is blocked. Figure B represents the schematic diagram of AF recurrence after PFA, as the direct death area doesn't cover the epicardium due to improper operation or the patient's own condition during the operation, the situation shown in Figure B will occur, and the PV electrial signal is not isolated, as well as AF will recur. In general, normal patients will directly go through stage A after PFA, while AF recurrence patients will exprience stage B first and then type A.

This study is based on the situation we found in the clinical trial. The time course of myocardial cell apoptosis obtained in the experiment is not only consistent with the phenomenon we observed in the clinic, but also consistent with the conclusion of the left ventricular apoptosis experiment of Zhao et al. (12). in rabbits. According to our research on cardiomyocyte apoptosis, analysis of HE staining results, clinical situation and research on PFA literature (2, 5, 10, 18, 19), the following deductions are made: In the central ablation area close to the electrode with high field strength and current density, PFA causes immediate cell death, resulting in the formation of a complete isolation zone. This is reflected clinically by the mapping result showing complete isolation. However, in the edge of ablation area with weaker field strength and current density, EP is insufficient to cause immediate cell death and PVI (Figure 10B). Other than that, myofibroblasts began to appear in the dead area of myocardial cells and myocardial intima, it will form re-entry circuit (11) and result in the recurrence of Af in the early stage of ablation (11, 19). The number of fibroblasts reaches its peak at 7 days and gradually decreases over the next 30 days, while fibrosis does not decrease. This is believed to be due to the transformation of myofibroblasts into fibrocytes (10). However, as the myocardial cells that did not die immediately undergo gradual apoptosis, just like we found that the peak of cardiomyocyte apoptosis occurs around 3 days after the procedure and results in the formation of a permanent isolation zone (Figure 10A), Af heals spontaneously and does not recur.

Unlike cell apoptosis caused by PFA, thermal ablation methods such as radiofrequency and cryoablation primarily result in cell necrosis (13). Post-operative fibrosis is primarily due to inflammation cells (5).

Based on the conclusions drawn, we propose the following ways to improve the clinical outcomes of PFA: Firstly, we consider that instances of AF recurrence are within the expected range of PFA and can be managed through medication, without the need for additional ablation or supplementary ablation. Secondly, we recommend extending the post-PFA observation period. Thirdly, enhancing the ablation catheter and strategy may bring about positive changes in PFA's clinical application. Lastly, with a better understanding of PFA and increased surgical experience, it is anticipated that these issues will be effectively addressed.

The parameters and catheters used in our animal and clinical trials were based on a large body of previous animal and cell experiments (1, 2). We used biphasic asymmetric pulses, which have been shown to have advantages such as lower ablation thresholds and less muscle contraction (1, 14–16). However, the timing of apoptosis may vary with different pulse parameters or catheters due to the existence of grading effects (9). Despite this, the overall time-related trend is expected to remain unchanged. Thus, we believe that our experimental conclusions are informative for the entire PFA procedure.

## Limitations

This study was unable to provide specific data regarding the ongoing clinical situation reported in the article due to confidentiality restrictions. The experiment was simplified by not considering the effect of animal sex and age on the results. The sample size limitation also prevented the accurate measurement of the time node of apoptosis and instead, the study only measured the daily changes in apoptosis. The results provide a macroscopic observation of the trend of apoptosis, but more detailed and grouped studies are needed to more accurately measure the subtle changes in apoptosis.

## Data Availability

The original contributions presented in the study are included in the article/Supplementary Material, further inquiries can be directed to the corresponding author/s.

## References

[1] Ye X Liu S Yin H He Q Xue Z Lu C Study on optimal parameter and target for pulsed-field ablation of atrial fibrillation. Front Cardiovasc Med. (2021) 8:690092. 10.3389/fcvm.2021.690092 34621795 PMC8490619

[2] Bi S Jia F Lv C He Q Xu X Xue Z Preclinical study of biphasic asymmetric pulsed field ablation. Front Cardiovasc Med. (2022) 9:859480. 10.3389/fcvm.2022.859480 35402543 PMC8987372

[3] Reddy VY Anic A Koruth J Petru J Funasako M Minami K Pulsed field ablation in patients with persistent atrial fibrillation. J Am Coll Cardiol. (2020) 76(9):1068–80. 10.1016/j.jacc.2020.07.007 32854842

[4] Reddy VY Koruth J Jais P Petru J Timko F Skalsky I Ablation of atrial fibrillation with pulsed electric fields: an ultra-rapid, tissue-selective modality for cardiac ablation. JACC Clin Electrophysiol. (2018) 4(8):987–95. 10.1016/j.jacep.2018.04.005 30139499

[5] Koruth J Kuroki K Iwasawa J Enomoto Y Viswanathan R Brose R Preclinical evaluation of pulsed field ablation: electrophysiological and histological assessment of thoracic vein isolation. Circ Arrhythm Electrophysiol. (2019) 12(12):e007781. 10.1161/CIRCEP.119.007781 31826647 PMC6924932

[6] Jiang C Davalos RV Bischof JC. A review of basic to clinical studies of irreversible electroporation therapy. IEEE Trans Biomed Eng. (2015) 62(1):4–20. 10.1109/TBME.2014.2367543 25389236

[7] Wojtaszczyk A Caluori G Pesl M Melajova K Starek Z. Irreversible electroporation ablation for atrial fibrillation. J Cardiovasc Electrophysiol. (2018) 29(4):643–51. 10.1111/jce.13454 29399927

[8] Rubinsky B Onik G Mikus P. Irreversible electroporation: a new ablation modality—clinical implications. Technol Cancer Res T. (2007) 6(1):37–48. 10.1177/153303460700600106 17241099

[9] Zager Y Kain D Landa N Leor J Maor E. Optimization of irreversible electroporation protocols for in-vivo myocardial decellularization. PLoS One. (2016) 11(11):1–15. 10.1371/journal.pone.0165475 27893744 PMC5125564

[10] Koruth JS Kuroki K Iwasawa J Viswanathan R Brose R Buck ED Endocardial ventricular pulsed field ablation: a proof-of-concept preclinical evaluation. Europace. (2020) 22(3):434–9. 10.1093/europace/euz341 31876913 PMC7058968

[11] McArthur L Chilton L Smith GL Nicklin SA. Electrical consequences of cardiac myocyte: fibroblast coupling. Biochem Soc Trans. (2015) 43(3):513–8. 10.1042/BST20150035 26009200

[12] Zhao Z Chen Y Wu B Qiu G Hong L Chen X Study of necrotic apoptosis by pulsed electric field ablation in rabbit left ventricular myocardium. Front Cardiovasc Med. (2022) 9:1012020. 10.3389/fcvm.2022.1012020 36225956 PMC9548611

[13] McBride S Avazzadeh S Wheatley AM O'Brien B Coffey K Elahi A Ablation modalities for therapeutic intervention in arrhythmia-related cardiovascular disease: focus on electroporation. J Clin Med. (2021) 10(12):1–20. 10.3390/jcm10122657 34208708 PMC8235263

[14] Cvetkoska A Macek-Lebar A Trdina P Miklavcic D Rebersek M. Muscle contractions and pain sensation accompanying high-frequency electroporation pulses. Sci Rep-Uk. (2022) 12(1):1–15. 10.1038/s41598-022-12112-9 PMC9110404 35577873

[15] Napotnik TB Polajzer T Miklavcic D. Cell death due to electroporation—a review. Bioelectrochemistry. (2021) 141:1–18. 10.1016/j.bioelechem.2021.107871 34147013

[16] Sano MB Fan RE Xing L. Asymmetric waveforms decrease lethal thresholds in high frequency irreversible electroporation therapies. Sci Rep. (2017) 7:40747. 10.1038/srep40747 28106146 PMC5247773

[17] Davalos RV Mir IL Rubinsky B. Tissue ablation with irreversible electroporation. Ann Biomed Eng. (2005) 33(2):223–31. 10.1007/s10439-005-8981-8 15771276

[18] Reddy VY Neuzil P Koruth JS Petru J Funosako M Cochet H Pulsed field ablation for pulmonary vein isolation in atrial fibrillation. J Am Coll Cardiol. (2019) 74(3):315–26. 10.1016/j.jacc.2019.04.021 31085321

[19] Atienza F Jalife J. Reentry and atrial fibrillation. Heart Rhythm. (2007) 4(3):S13–S6. 10.1016/j.hrthm.2006.12.004 17336877

